# A novel bio-psycho-social approach for rehabilitation of traumatized victims of torture and war in the post-conflict context: a pilot randomized controlled trial in Kosovo

**DOI:** 10.1186/s13031-016-0100-y

**Published:** 2017-02-08

**Authors:** Shr-Jie Wang, Ardiana Bytyçi, Selvi Izeti, Melita Kallaba, Feride Rushiti, Edith Montgomery, Jens Modvig

**Affiliations:** 1 Danish Institute against Torture (DIGNITY), Copenhagen, Denmark; 2 Kosova Rehabilitation Centre for Torture Victims (KRCT), Pristina, Kosovo; 3 Department of Psychology, University of Pristina, Pristina, Kosovo

## Abstract

**Background:**

Some evidence showed that multidisciplinary rehabilitation in Western countries is effective for treating war-related trauma, but it remains unclear whether this approach is applicable to civilians living in resource-poor countries affected by war. In 2012–14, Danish Institute against Torture (DIGNITY) conducted a randomized controlled trial (RCT), in partnership with Kosova Rehabilitation Centre for Torture Victims (KRCT), to examine the effects of multidisciplinary intervention among victims of torture and war in Kosovo.

**Methods:**

A single-center, randomized, parallel-arm, single-masked, waiting-list controlled trial was implemented in northern Kosovo. Thirty-four participants meeting the recruiting criteria were randomized to either intervention group, which received integrated treatments plus a once-daily multivitamin, or the waiting list group, which received multivitamin alone. The integrated treatments consisted of 10 weekly individual 60-min sessions of cognitive behavioral therapy (CBT), based on an adapted prolonged exposure therapy manual, an individual 20-min breathing exercise with an emWave biofeedback device, and 90-min group physiotherapy. The waiting list group also received the same treatment after the intervention group had completed their sessions. Outcome assessments were conducted at 3, 6 and 9 months after baseline assessment. Outcomes measures consisted of 4 subtypes: mental, emotional, physical health, functioning and social outcomes, i.e. PTSD, depression, anxiety, chronic pain, anger and hatred expression, body mass index, handgrip strength, standing balance, income, employment rate and disability score.

**Results:**

Over 1/3 of PTSD cases were successfully treated. Inconsistent patterns with mental health and chronic pain outcomes were observed while there was a definite impact of intervention on functioning and social outcomes, i.e. the employment rate, which increased nearly 15 %, and the monthly wage, which rose 45–137 %. There was also a noticeable improvement in handgrip strength and disability score; the feelings of anger and hatred diminished. However, most of these changes did not reach statistical significance.

**Conclusions:**

The impact of bio-psycho-social intervention is likely sensitive to the context of post-war economy in Kosovo and the treatment goals. The potential for improving the emotional well-being and employment outcome in victims was demonstrated. A larger scale RCT in a similar setting is needed, with close monitoring of treatment integrity and data reliability.

**Trial registration:**

Clinicaltrials.gov (NCT01696578).

## Background

Studies have demonstrated the long-lasting effect of ethnic conflict on the health and well-being of the Kosovar population [1–5]. The Danish Institute against Torture (DIGNITY), formerly Rehabilitation and Research Centre for Torture Victims, has been involved since 1999 in a number of research and intervention projects among victims of torture and war in Kosovo, in partnership with the Kosova Rehabilitation Centre for Torture Victims (KRCT) [1, 6, 7]. In our previous papers, we found that the level of lifetime exposure to massive violence, the proportion of family members reporting pain and injury related to lifetime violence, and the family’s financial burden were inter-correlated. Possible factors contributing to healing at individual, family and community levels were investigated. There was a substantial overlap of chronic pain and multiple comorbid conditions. The results also showed that feelings of anger and hatred, military or police phobia, and an inferiority complex amplify pain experience, and their interactive effects contribute to poorer physical condition and sleep quality among the affected population in Kosovo. The problems suffered by victims often reduce their ability to cope with day-to-day activities and to hold jobs.

There have been many types of health interventions to improve the situation of adult survivors of torture or war trauma, and for many of these the outcome has been assessed and discussed [8]: the authors found less than 10 randomized controlled trials (RCTs) or quasi-RCTs, and many studies did not systematically address a frequent occurrence of comorbidity with other symptoms, nor did they examine other important dimensions such as quality of life or functionality that affected people’s everyday life. Another review paper examined 30 years of research evidence about interventions [9] and found that they generally showed a fair outcome in terms of general psychopathology in spite of variability in study design. However, the review also highlighted the bias that most of the interventions evaluated were in Europe and North America and not among people still living in the country where they had been exposed to war or massive violence.

There is a need to continue interventions in northern Kosovo among those who have long-lasting problems following the exposure to trauma. Therefore, in 2012–2014 DIGNITY in collaboration with KRCT carried out a RCT to examine the effects of multidisciplinary intervention on comorbid chronic pain and affective disorders, physical conditions, emotional well-being, coping and functioning. We used multidisciplinary approach that has shown its benefit especially for comorbid pain and mental disorders among general population [10–13] and among people affected by torture or war living in Europe or North America [9, 14–17].

It is a first pilot study which addresses the knowledge gap about the feasibility and the effectiveness of bio-psycho-social approach for a population exposed to torture and war, living in a resource-poor country. The bio-psycho-social approach in our intervention in Kosovo is based on the concepts underlying DIGNITY’s multidisciplinary rehabilitation model and lessons learnt from DIGNITY’s experience in Denmark over 30 years [18]. The main objective was to test the effects of a multidisciplinary, short-term treatment intervention with victims of torture or war related trauma in Kosovo, using instruments from various perspectives.

## Methods

### Trial design

**Fig. 1:**
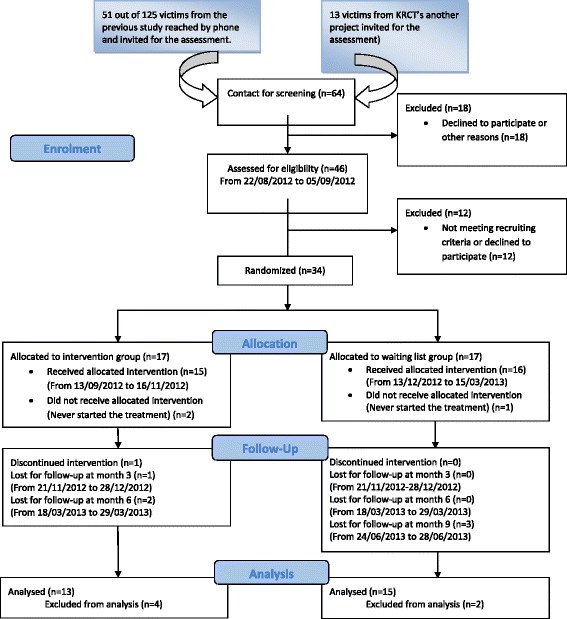
CONSORT flow diagram for enrolment

This was a single-center, randomized, parallel-arm, single-masked, controlled trial to examine the value of a variety of interventions for a traumatized population in northern Kosovo. Thirty-four participants were all victims of torture or war-related trauma, who were divided randomly into two groups, “intervention” and “waiting list”. Recruitment and baseline assessment was undertaken in August and September 2012 (month 0). Outcome assessments were carried out at months 3, 6, and 9 (Fig. 1).

### Sample size

We calculated that a minimum of 17 participants in each arm of the trial was required. Our forecast was principally based on the findings of a study in Kosovo [19], therefore, the sample size was calculated for the detection of a 0.4 deduction in the mean score of Harvard Trauma Questionnaire (HTQ), with power of 90 % (1 − *β* error probability) and statistical significance of *P* value <0.05.

### Participants and enrolment

A two-phase procedure was carried out to identify eligible participants. In the first phase, we identified 51 potential participants who had previously been screened in a population-based study [1, 6, 7] and 13 potential participants from the neighboring areas who had been involved in another KRCT project. In total, 64 were contacted and invited to take part in an initial screening for the RCT. They were provided with brief information about the planned treatment package. In the second phase, the 64 preliminary selected individuals were screened for eligibility by an independent Kosovo psychiatrist who was not working with either DIGNITY or KRCT. Participants were required to be 18–65 years old and to have reported one or more of the following experiences: 1) torture and other cruel, inhuman or degrading treatment or punishment, using UN definition; 2) sexual harassment, molestation, rape or insertion of a blunt object into a genital organ and/or the rectum; 3) arrest and detention without warrant or order; or 4) extrajudicial execution of family members, perpetrated by members of law enforcement agency. All the participants should meet the Diagnostic and Statistical Manual of Mental Disorders (DSM-IV) criteria of comorbid chronic pain and one of the affective disorders: post-traumatic stress disorder (PTSD), depression or anxiety. People with the following conditions were excluded: 1) mental retardation or significant speech or cognitive impairment that would impede assessments, 2) past or present schizophrenia, 3) major alcoholic or substance abuse problems. Patients were also excluded if they had recently undergone chemotherapy or chemo-radiotherapy for cancer or were going to have such therapy within following 6 months, or if they had undergone any CBT in the past three years.

During the interviews, information about the study design, procedures and therapy was given. After having given their consent to the trial, 34 patients confirmed their participation. Due to logistic constraints and quality concerns, KRCT had difficulty in getting more participants into the trial and decided to stop the recruitment when there were 34 participants. The CONSORT flow diagram demonstrates the details of recruitment and retention in the trial (Fig. 1).

### Randomization and masking

The 34 participants were randomly allocated to the intervention group or the waiting list group by a block randomization procedure using a computerized random number generator by two blocks of size 17, created by a DIGNITY staff not involved in the trial. Each participant was given a unique number. The participants in each group were randomly assigned to three therapists, using a block randomization technique performed by another DIGNITY staff not involved in the trial. At the baseline assessment, the participants and therapists were blinded to the allocation. The therapists were also blinded to the outcomes during the baseline and outcome assessments. The baseline and outcome assessor was blinded to which group was which, throughout the study.

### Intervention

The multidisciplinary intervention was provided according to a study protocol prepared by the first author. The treatment included 10 individual therapy sessions, using biofeedback supported CBT (BF-CBT) and 10 group therapy sessions (physiotherapy and exercises) on a weekly basis over a 3-month period. Individual sessions lasted on average 90 min, and group exercise sessions were from 60 to 90 min in length. In order to improve physical and emotional well-being, the participants were provided with two bottles of multivitamin (Centrum® Adult: 90 tablets) for daily intake for a period of 6 months immediately after the baseline. Some participants in the waiting list group showed signs of aggressiveness or frustration because they would have to wait for 3 months. The multivitamin was therefore given to both groups at the same time, as it aimed to prevent further drop-out and it would have been unethical to give only a placebo to waiting list group from a traumatized population.

#### Individual sessions

Each BF-CBT session lasted 90 min, including a 60-min CBT intervention and a 15–20 min period of breathing training using a biofeedback device, plus 10–15 min for reviewing and note-taking purposes together with the participant*.* CBT interventions were mainly based on an adaptation of the prolonged exposure therapy (PET) manual originally written by Foa et al. that focuses on addressing trauma-related fears and symptoms. The manual allows for some flexibility in the use of PET; for example, the treatment can be modified with respect to the number and length of sessions [20–26]. The PET protocol was adapted by the DIGNITY psychologist Uwe Harlacher and KRCT team members so that it could be applied in a resource-poor setting. Both agreed that 10 sessions would be workable for therapists and patients in Kosovo. The key element was exposure to trauma memories. A minor element was psycho-education and anger management. There were three main procedures for each session. 1) Education about common reactions to trauma. 2) Imagined (repeated) exposure to the trauma memories. The theory underlying repeated exposure is that the participants repeatedly approach and then get relief from the traumatic situation. Confrontation with the traumatic experiences should enhance the processing of these experiences and modify dysfunctional cognitions. 3) There is also a homework assignment in the original manual but this component was omitted since both the therapists and participants felt confused, stressed and burdened with the homework tasks*.*

Breathing re-training used a heart rate variability (HRV) biofeedback device, and was accompanied by the therapists. Heart and breathing rhythms may accelerate in a panic attack when patients confront trauma-related imagery. Recent studies have reported positive results when biofeedback, helping patients to control HRV, is added to CBT in patients with panic disorder [27] and especially those with PTSD [28, 29]. Exhalation is associated with relaxation. The participants were helped to slow down breathing for 15–20 min by learning to control their own heart and breathing rhythms to avoid hyperventilation. A small sensor linked to the biofeedback device was clipped on to a finger or the earlobe of a participant while he or she was practicing breathing. Breathing and HRV were displayed in a dynamic, user-friendly graphics on the screen of a laptop with a biofeedback programme (HeartMath® emWave2: HeartMath Institute, 2012). When the participant and the therapist could see the patient’s breathing pattern over time, therapists could check whether adjustment was needed. The participants were asked to do breathing exercises at home without using a biofeedback device when they experienced emotional disturbance.

#### Group sessions

The participants were asked to take part in group therapy once a week. It normally took place on the same day as BF-CBT sessions. The participants gathered together in a big room and a series of physical games and activities for the group were introduced to them by a physiotherapist to enhance their physical activity and participation level. Each exercise session included warming-up exercise. The physiotherapist also engaged the participants in activities that facilitated communication, in which they worked together as a team. Each group session lasted 60–90 min; the length of exercise was adjusted according to the capacity and condition of the participants. For example, people who felt exhausted or overwhelmed after exposure therapy could go home earlier. Gradually, to increase their activity level, participants were encouraged to walk from the main bus station in Pristina to the KRCT office before the treatment and return to the bus station from the KRCT office on foot afterwards. The walk took around 20 min each way.

### Outcome assessments

Outcomes variables comprised of four subtypes: mental health, emotional well-being, physical health, functioning and social outcomes. The mental health outcome measures were PTSD, depression and anxiety symptoms. The emotional well-being outcomes were the reporting of anger, aggressiveness, inferiority complex, social isolation and police or military phobia within 14 days. Physical health outcome measures were chronic pain symptoms, body mass index (BMI), handgrip strength and standing balance. The functioning and social outcomes were income, employment rate, and disability score.

Baseline assessment was undertaken for both groups from 22 August to 5 September, before the intervention started on 13 September, 2012. The waiting list group also received the same treatment on 13 December, 2012 after the intervention group had completed their sessions. For the intervention group the outcome assessment took place immediately at the end of treatment in November 2012 (month 3) and at follow-up in March 2013 (month 6) and for the waiting list group in November 2013 (month 3), just before they started treatment, and at the end of treatment in March (month 6) and at follow-up in June 2013 (month 9), respectively. The task was carried out by an external assessor who also conducted the initial screening. Non-responders were reminded by telephone to participate in the assessments according to prior consent (Fig. 1).

The assessment used a set of self-reported standard questionnaire (see the details below) for a structured interview, with a general checklist to collect data on demography, trauma and medication history and emotional problems. The general checklist also included pain frequency during the past week and pain location. Pain location was self-reported and was recorded on a drawing outlining a male or a female figure: the Margolis Pain Diagram [30]. Physical examinations were conducted alongside the interview. A nested qualitative interview was also conducted at month 6 for the intervention group and month 9 for the waiting list group. The KRCT team studied these raw materials using a qualitative approach; the results will be reported elsewhere.

All the standard questionnaires were translated from English to Albanian and back-translated. Except for questionnaire 5, questionnaire 1–4 (see the details below) had been validated for use in Kosovo by the CDC [4, 31], or as part of the earlier collaboration between KRCT and Danish Refugee Council [5] and DIGNITY [1, 6].

#### Standard self-reporting questionnaires

1. Harvard Trauma Questionnaire (HTQ)-part II
2. Hopkins Symptoms Checklist-25 items (HSCL-25)
3. Short-form McGill Pain Questionnaire (SF-MPQ)
4. Wong-Baker FACES® Pain Rating Scale
5. World Health Organization Disability Assessment Schedule 2.0 (12 items) (WHODAS 2.0)

In this study, internal consistencies of the HTQ-part II, HSCL-25, SF-MPQ and WHODAS 2.0 (12 items) were examined using Cronbach’s alphas.

The HTQ is a questionnaire with 30 items to assess PTSD. It combines a list of potentially traumatic events (part 1) and symptoms of PTSD (part 2) selected from the DSM-IV. The cut-off value for case status of HTQ is 2.5. HTQ was translated into Kosovar Albanian and back translated into English by the CDC [4, 31]. Dr. Barbara Lopes Cardozo adapted the trauma event questions based on a rapid qualitative assessment in 1999, and used the 16 PTSD symptom questions, which follow the DSM-IV criteria. The Albanian version of HTQ has been used in many studies in Kosovo since then [5, 32, 33]. The Cronbach’s alpha for the total score of HTQ-part II in this study is *α* = 0.87, which is good.

The HSCL-25 is a 25 item self-reporting symptom inventory for depression and anxiety disorders. The cut-off value for case status is 1.75. HSCL-25 has been proven to be internally consistent and valid for different refugee groups and war survivors. It has been validated by translation-back translation from English to Kosovar Albanian and used in the 2006 and 2009 studies [5, 34]. In the present study it showed high internal stability (HSCL-25 depression subscale, *α* = 0.91 and anxiety subscale, *α* = 0.88, respectively).

For pain assessment, the Wong-Baker FACES® Pain Rating Scale and the SF-MPQ were used. They have been widely proven to be valid instruments capable of providing information about pain severity and intensity [7, 35–37]. In the absence of a gold standard for pain, the criterion validity of Wong-Baker FACES® Pain Rating Scale cannot be evaluated, while some studies tested the reliability and validity of this scale for chronic pain in children [38, 39]. The Wong-Baker FACES® Pain Rating Scale uses cartoon smiling face indicating “no pain” to tearful face indicating “worst pain” to gauge pain intensity in patients who cannot communicate well. The SF-MPQ has been translated into different languages and some studies have shown the validity of the translated versions [36]. Translation back-translation of the English version of the SF-MPQ was done independently by a translation firm and adapted in the earlier study in 2008–2010 [6]. The Kosovar Albanian version of the SF-MPQ is a reliable and valid instrument for the measurement of pain in Albanian speaking patients with chronic pain and comorbid mental disorders, with the Cronbach’s alpha of 0.90.

“WHODAS 2.0” is a simple, reliable and valid questionnaire that measures self-reported functioning and quality of life. Its results are compatible with the system of international classification of functioning. It treats all disorders at parity when determining the level of functioning. The short version contains 12 questions assessing functional impairment in the areas of work*,* home management, social life, physical activity and close relationships. The WHODAS 2.0 has already been translated into 31 languages, including Albanian, following a rigorous WHO translation and back-translation protocol [40]. A series of field studies was used to test the cross-cultural applicability, reliability and validity, as well as the utility of WHODAS 2.0 in health service search by WHO and its partners [41]. It was the one instrument that had not been used in our previous studies. The WHODAS 2.0 (12 items) gives a Cronbach’s alpha of 0.88, also showing a good internal consistency in this present study.

#### Physical examinations

1. BMI
2. Handgrip strength
3. Standing balance test

Physical examinations were conducted in order to provide an objective measurement to supplement the subjective results expressed in the oral reports. To assess physical fitness, BMI was calculated, and muscle strength and standing equilibrium were tested. The procedures have been described elsewhere [6, 42]. To calculate BMI, height and weight measurements were made in a standing position without shoes. We assessed muscle strength by measuring handgrip strength with a Jamar® hydraulic hand dynamometer according to the procedure of the American Society of Hand Therapists [43]. The ability to maintain physical equilibrium was assessed by a standard standing balance test. If the participants could keep their balance on one leg for more than 30 s, this was considered a successful outcome [44].

### Team composition

The research team consisted of an epidemiologist, three therapists (one medical doctor and two psychologists), three physiotherapists, two clinical supervisors from DIGNITY (CBT experts) and one external assessor from Kosovo for the baseline and outcome assessments, as well as an external assessor from Israel to evaluate the treatment integrity and compliance. The two psychologists studied at postgraduate level at the University of Pristina and the medical doctor specialized in mental health and had some CBT experience. The therapists’ working clinical experience ranged from 1 to 15 years. The three physiotherapists also had university degrees and few years of working experience.

### Quality assurance

The study design and the methodology of this project were introduced to the team members by the first author. All therapists attended a one-week training workshop and two refresher courses where a clinical supervisor from DIGNITY, Uwe Harlacher, taught them the principles and implementation of the standard PET. Throughout the trial, two-hour long-distance supervision was provided weekly by two clinical supervisors (Uwe Harlacher and Sadia Khan) at DIGNITY using Skype meetings. Supervision included discussion of individual cases, therapy in progress, barriers, clarification and adjustment of treatment protocols, and cultural issues. The supervision also aimed to ensure that the therapists were all following the therapy protocol in the same way. Additionally, to check treatment integrity and compliance for quality assurance, at least 15 % of the CBT treatment sessions were randomly selected and recorded using a digital audio recorder. An independent assessor from Israel, who is a visiting fellow at the Department of Psychology at University of Pristina, reviewed these recorded sessions with the assistance of an experienced interpreter with medical doctor background. DIGNITY physiotherapy manual was provided to the physiotherapists for the group exercise, but the group therapy sessions were not monitored closely.

The CONSORT checklist and flow diagram for RCT were followed to ensure the reporting quality [45].

### Data analysis

An intention-to-treat analysis was used. The dataset was entered in the Statistical Package for the Social Sciences (SPSS™, Windows version 12) and converted into STATA version 13.0 (StataCorp LP, Texas, USA, 2013) for data cleaning and analysis. The dataset was validated after the discrepancies between the original paper forms and the dataset had been corrected. Missing values were treated according to the manual instruction. Missing values for the outcomes in the PTSD, depression, anxiety disorder, pain rating index and disability scales were replaced with the median value for the corresponding item across both intervention and waiting list groups before calculating the average score across all items. If all items are missing for any participant then that participant is not included in the analysis (this affected two individuals with missing SF-MPQ pain rating scores at 9 months, one individual with missing bodily pain sites at 9 months, and one individual with missing disability score at baseline). Socio-demographic characteristics and baseline variables in both groups were tested using Fisher’s exact test for categorical variables and the Wilcoxon rank sum for continuous variables to assess their comparability. Pain locations were grouped into six major categories as we did in a previous study [1]: head, neck/shoulder, chest/abdomen, back, upper limb and lower limb, and the total number of sites was recorded. Pain intensity and frequency were dichotomized to maintain reasonable numbers in each group. Therefore, pain intensity was recoded as no pain/light pain versus moderate/severe pain and pain frequency was recoded as occasional/periodic versus consistent pain, since all of the participants reported some pain experience.

We used multivariable regression models to estimate intervention effects, focusing on change from baseline with adjustment for baseline to minimize bias. One regression model was to compare intervention with nothing at month 3 (immediately after the treatment for the intervention group); and another one was to compare longer term effect with shorter term effect, at month 6 (immediately after the treatment for the waiting list group). We also ran an additional multivariate regression analysis to compare the intervention and waiting list groups 3 months after the end of the intervention, i.e. in the intervention group we presented outcomes at 6 months adjusted for differences at 3 months and in the waiting group we present outcomes at 9 months adjusted for differences at 6 months. None of the participants was taking medication at the baseline, and they were informed that they must avoid taking any medication throughout the trial. However, it was found that around half of participants did start taking medications for PTSD, depression, anxiety disorder or chronic pain, at different time points during the intervention. This was largely consistent between the two groups (Table 1). Medication use was additionally adjusted in the multivariate regression models. It was not ethically acceptable to ask the participants to stop taking the medication.

**Table 1:** Characteristics of intervention and waiting groups at baseline

	Waiting (*N* = 15)	Intervention (*N* = 13)	p*
*N* (%)			
Male	9 (60.0)	6 (46.2)	0.71
Female	6 (40.0)	7 (53.9)	
Married	11 (78.6)	11 (84.6)	1.00
Unmarried^a^	3 (21.4)	2 (15.4)	
Primary school only	7 (50.0)	7 (53.9)	1.00
More than primary school^a^	7 (50.0)	6 (46.1)	
Unemployed/household work	10 (66.7)	12 (92.3)	0.17
Employment	5 (33.3)	1 (7.7)	
Mitrovica district	3 (20.0)	2 (15.4)	0.95
Skendervej district	6 (40.0)	4 (30.8)	
Vushittri district	1 (6.7)	2 (15.4)	
Other districts	5 (33.3)	5 (38.5)	
No PTSD	9 (60.0)	5 (38.5)	0.45
PTSD (cut-off value:2.5)	6 (40.0)	8 (61.5)	
No depression	2 (13.3)	0 (0.0)	0.48
Depression (cut-off value:1.75)	13 (86.7)	13 (100.0)	
No anxiety	1 (6.7)	2 (15.4)	0.58
Anxiety (cut-off value:1.75)	14 (93.3)	11 (84.6)	
No/light pain	2 (14.3)	4 (20.8)	0.39
Moderate/severe pain	12 (85.7)	9 (69.2)	
No/occasional/periodic pain	8 (57.1)	9 (69.2)	0.70
Consistent pain	6 (42.9)	4 (30.8)	
No medication at month 3	6 (40.0)	6 (46.2)	1.00
Any medication at month 3	9 (60.0)	7 (53.9)	
No medication at month 6	7 (46.7)	6 (46.2)	1.00
Any medication at month 6	8 (53.3)	7 (53.9)	
No medication at month 9	NA	5 (38.5)	-
Any medication at month 9	NA	8 (61.5)	
*Mean (Standard deviation: SD)*			
Age^a^	48.8 (10.9)	46.8 (10.4)	0.70
Monthly income in Euro^b^	116.2 (106.7)	113.5 (75.7)	0.66
PTSD score	2.4 (0.4)	2.5 (0.5)	0.66
Depression score	2.5 (0.7)	3.0 (0.6)	0.03
Anxiety score	2.4 (0.6)	2.6 (0.7)	0.38
SF-MPQ Pain Rating Index	1.0 (0.7)	1.5 (0.7)	0.07
SF-MPQ Affective Pain Score	1.1 (0.7)	1.5 (0.6)	0.13
SF-MPQ Sensory Pain Score	0.6 (0.8)	1.4 (1.0)	0.03
Number of bodily pain sites	3.3 (1.3)	2.8 (1.2)	0.30
Body Mass Index (BMI) ^a^	27.9 (4.1)	27.6 (7.2)	0.56
Standing balance right foot (seconds) ^a^	52.0 (34.1)	47 (30.9)	0.96
Standing balance left foot (seconds) ^a^	46.5 (35.5)	47.0 (33.8)	0.88
Grip strength right hand (kg)	31.3 (13.0)	29.0 (12.9)	0.47
Grip strength left hand (kg)	28.6 (17.6)	23.6 (12.2)	0.49
WHODAS 2.0 score^a^	1.4 (0.7)	2.1 (0.8)	0.02

^a^Missing for at least one respondent; ^b^Too many missing respondents at the baseline: waiting list - 6 and intervention - 10, *p value from Fisher’s exact test for categorical outcomes, Wilcoxon’s rank sum test for continuous outcome

## Results

Two participants never began the treatment and one dropped out before the 2nd session was completed. Some outcome values were missing systematically from 6 participants at the month 6. In total, 13 participants in the intervention group and 15 in the waiting list group were included in an intent-to-treat analysis (Fig. 1). A post-hoc power analysis identified a power of 77 % for a final sample size of 28 participants, in detecting an effect PTSD.

In total, 244 individual treatment sessions were provided to 32 participants in both groups. The average attendance rate for the individual therapy sessions in the two groups was satisfactory, around 76 %. The attendance rate for group therapy sessions was 55 %. The attendance rate was higher for the waiting list group but there was no significant difference between both groups for the treatment session attendance.

Table 1 summarizes the socio-demographic and baseline characteristics, which are similar in both groups. There were slight differences for the mean scores of depression, SF-MPQ and WHODAS 2.0, which indicated that the participants in the intervention group had worse baseline symptoms and disability, although the participants with depression or chronic pain were equally distributed in both groups. Overall, the randomization seems to be acceptable in terms of generating two comparable groups with similar socioeconomic characteristics; however, morbidity was slightly greater in the intervention group.

The mean age for both groups taken together was 47.7 years old. There were 55.0 % men and 45.0 % women. Half of them had only primary school education. At the baseline, 21.4 % of participants in both groups were in paid employment (5 out of 15 men and 1 out of 13 women), and their mean monthly income was around 115 Euro (Table 1).

Clinical diagnosis and the standard questionnaire showed that all of the participants reported comorbid pain and at least one of affective disorders (PTSD, depression, anxiety disorders) at the baseline: 14 (50.0 %) met criteria for PTSD, 26 (92.9 %) for depression, and 25 (89.3 %) for anxiety disorders. The average number of pain sites reported on the Margolis Pain Diagram was 3 for both groups of the trial.

The average BMIs for both groups were higher than 27 kg/m^2^ (95 % CI: 25.2–29.3) which is the overweight category. Both groups showed good standing balance (>30 s) but poor handgrip strength.

**Fig. 2:**
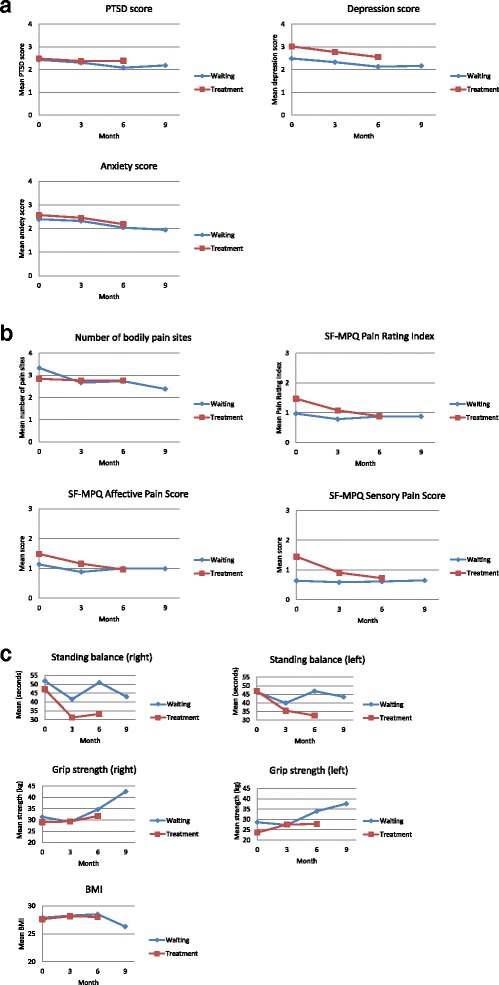
**a** Mean of mental health outcomes over time: PTSD, depression and anxiety disorders. **b** Mean of pain outcomes over time: numbers of bodily pain sites and SF-MPQ. **c** Mean of physical health outcomes over time: handgrip strength, standing balance and BMI

**Fig. 3:**
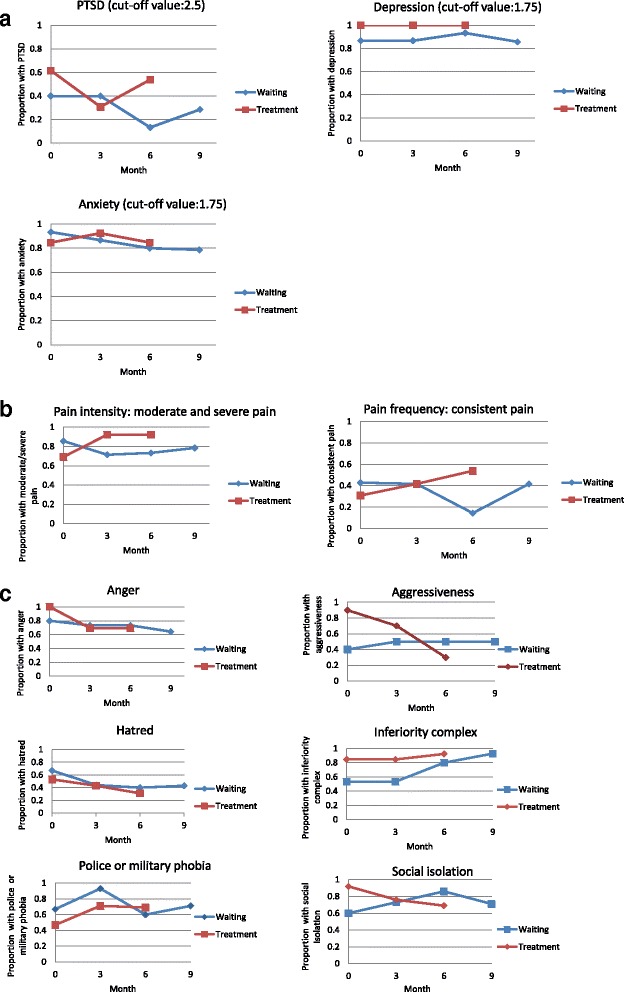
**a** Proportion of binary outcome over time: PTSD, depression and anxiety disorders. **b** Proportion of binary outcome over time: pain intensity and frequency. **c** Proportion of binary outcome over time: emotional well-being

**Fig. 4:**
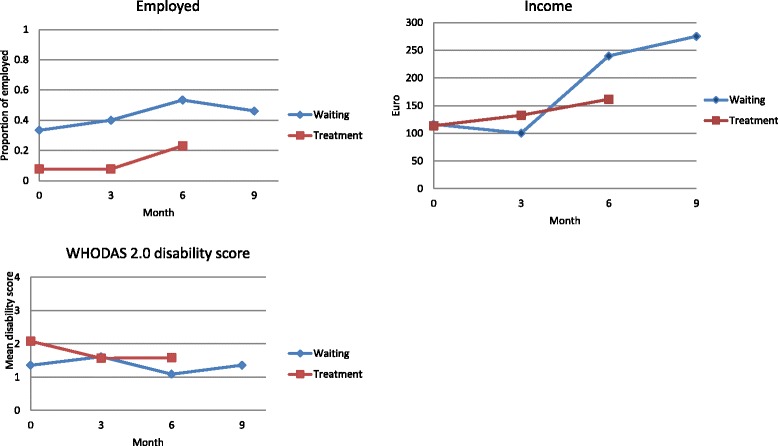
Functioning and social outcomes over time: employment rate, monthly income and disability score

Figures 2a-c, 3a-c and 4 display the changes over time in the continuous and binary outcomes for both groups. The employment rate as measured 3 months after the last treatment increased from 7.6 % at baseline to 23.1 % for the intervention group and from 33.3 % at baseline to 46.2 % for the waiting list group, respectively (Fig. 4). The mean monthly income also rose significantly within 6 months from 113 to 164 Euro (increase of 45.1 %) for the intervention group and 116 to 275 Euro (increase of 137.1 %) for the waiting list group, respectively. There was also an increase in handgrip strength in the right hand over the course of trial, from 29.0 to 31.7 kg for the intervention group and from 31.3 to 42.6 kg for the waiting list group, respectively (Fig. 2c). The same pattern was observed for the handgrip strength in the left hand. However, the standing balance became worse for both legs and for both groups.

r

With respect to emotional well-being, both groups showed a slight reduction in the proportion of the participants with “feelings of anger and hatred within the past 14 days”, 15–20 % in the intervention group and 20–25 % in the waiting list group, respectively (Fig. 3c). There was a remarkable reduction in the proportion with feelings of aggressiveness in the intervention group but there was a slight increase in the waiting list group.

The other outcome measures showed an inconsistent pattern over time. Three months following the last treatment for both groups, there were 9 participants who met criteria for PTSD, 27 for depression and 23 for anxiety disorders, respectively. The mean score for SF-MPQ Pain Rating Index was 1.1 at the baseline and 0.88 at the end of the trial. Figure 2a shows that there was a very small reduction of PTSD and anxiety disorders over time. At month 3 and month 6 the SF-MPQ Pain Rating Index seemed to be slightly lower in both groups (Fig. 2b). In addition, half the participants (8 out of 15) no longer reported a worse pain feeling at month 6.

Effects of intervention were assessed using multivariate analysis, as presented in the Tables 2 and 3. From pre-treatment to post-treatment and 3 months following the last treatment, both regression models showed some improvement in handgrip strength, and in feelings of anger and hatred, as well as income and employment. However, the change was not statistically significant. A short-term improvement in the overall WHODAS 2.0 scores was also observed at month 3, but the effect faded away at month 6, with one exception, “taking care of household”. In both regression models, there was little evidence of any substantial difference between the two groups; apart from that PTSD score might be higher in the intervention group than in the waiting list group at month 6. After the waiting list group had received treatment there were no longer significant differences in chronic pain and mental health outcomes at month 6, suggesting that the waiting list group made gains comparable to the intervention group. In general, power is low and this is a particular problem for the binary outcomes (Tables 2 and 3), where confidence intervals are generally wide and, in some cases, data were too sparse to complete the analyses.

**Table 2:** Impact of intervention on continuous outcomes at month 3 and 6 following baseline assessment

	Month 3			Month 6		
	*N* (Waiting/Intervention)	Difference (95 % CI^a^)		*N* (Waiting/Intervention)	Difference (95 % CI)	
		Adjusted for outcome at baseline	Additionally adjusted any medication use		Adjusted for outcome at baseline	Additionally adjusted any medication use
PTSD score	15/13	0.03 (−0.19, 0.26)	−0.03 (−0.25, 0.20) ^b^	15/13	0.27 (0.02. 0.53)	0.27 (0.03, 0.59) ^b^
*P*		0.76	0.80		0.04	0.08
Depression score	15/13	0.06 (−0.24, 0.35)	0.05 (−0.28, 0.39) ^c^	15/13	0.17 (−0.11, 0.45)	0.17 (−0.15, 0.50) ^c^
*P*		0.70	0.74		0.22	0.28
Anxiety score	15/13	0.04 (−0.22, 0.29)	−0.01 (−0.29, 0.28) ^d^	15/13	0.07 (−0.24, 0.38)	0.02 (−0.32, 0.36) ^d^
*P*		0.77	0.77		0.65	0.91
SF-MPQ Pain Rating Index	15/13	0.32 (0.03, 0.60)	0.32 (0.03, 0.61)	15/13	−0.03 (−0.28, 0.23)	−0.03 (−0.28, 0.23)
*P*		0.03	0.03		0.84	0.83
SF-MPQ Affective Pain Score	15/13	0.29 (0.02, 0.55)	0.28 (0.01, 0.55)	15/13	−0.06 (−0.33, 0.20)	−0.07 (−0.33, 0.20)
*P*		0.04	0.05		0.63	0.61
SF-MPQ Sensory Pain Score	15/13	0.40 (−0.05, 0.86)	0.43 (−0.03, 0.90)	15/13	0.13 (−0.31, 0.57)	0.14 (−0.30, 0.57)
*P*		0.08	0.07		0.56	0.52
Number of bodily pain sites	15/13	0.35 (−0.76, 1.47)	0.35 (−0.78, 1.47)	15/13	0.09 (−0.97, 1.16)	0.08 (−1.01, 1.17)
*P*		0.52	0.53		0.86	0.88
BMI	11/13	0.23 (−0.32, 0.78)	0.24 (−0.32, 0.81)	14/12	−0.27 (−0.91, 0.36)	−0.26 (−0.91, 0.39)
*P*		0.40	0.37		0.38	0.42
Standing balance right foot (seconds)	12/13	−5.02 (−27.68, 17.64)	−6.80 (−29.25, 15.64)	14/13	−11.49 (−32.66, 9.68)	−11.50 (−33.17, 10.17)
*P*		0.65	0.54		0.27	0.28
Standing balance left food (seconds)	12/13	−5.13 (−31.03, 20.78)	−8.30 (−32.80, 16.20)	14/13	−10.35 (−32.48, 11.77)	−10.79 (−32.85, 11.27)
*P*		0.69	0.49		0.34	0.32
Grip strength right hand (kg)	12/13	0.36 (−5.09, 5.82)	−0.68 (−5.29, 3.94)	15/13	−1.11 (−8.15, 5.94)	−1.12 (−7.40, 5.16)
*P*		0.89	0.76		0.75	0.72
Grip strength left hand (kg)	12/13	2.30 (−2.08, 6.69)	1.44 (−2.64, 5.52)	15/13	−0.95 (−5.82, 3.93)	−1.12 (−5.67, 3.43)
*P*		0.29	0.47		0.69	0.62
WHODAS 2.0 score	15/12	−0.20 (−0.83, 0.43)	−0.08 (−0.72, 0.56)	15/12	0.20 (−0.44, 0.84)	0.18 (−0.49, 0.86)
*P*		0.51	0.81		0.53	0.58

^a^ Confidential interval

^b^Adjusted with use of PTSD medication, ^c^Adjusted with use of depression medication, ^d^Adjusted with use of anxiety medication

**Table 3:** Impact of intervention on binary outcomes at month 3 and 6 following baseline assessment

	Month 3			Month 6		
	*N* (Waiting/Intervention)	Odds ratio (95 % CI)		*N* (Waiting/Intervention)	Odds ratio (95 % CI)	
		Adjusted for outcome at baseline	Additionally adjusted for any medication use		Adjusted for outcome at baseline	Additionally adjusted for any medication use
Anger	15/13	0.45 (0.07, 3.07)	0.46 (0.07, 3.15)	15/13	0.75 (0.13, 4.36)	0.76 (0.13, 4.47)
*P*		0.42	0.43		0.75	0.76
Aggressiveres	15/13	1.25 (0.21, 7.20)	1.44 (0.21, 10.07)	15/13	0.09 (0.01, 1.05)	0.09 (0.01, 1.08)
*P*		0.81	0.70		0.05	0.06
Hatred	15/13	0.83 (0.18, 3.89)	0.85 (0.18, 4.00)	15/13	0.81 (0.14, 4.61)	0.81 (0.14, 4.6)
*P*		0.82	0.83		0.82	0.82
Inferiority complex	15/13	2.90 (0.40, 22.29)	20.90 (0.38, 21.90)	15/13	0.75 (0.03, 17.51)	^a^
*P*		0.29	0.30		0.86	
Military or police phobia	15/14	0.15 (0.01, 2.16)	0.14 (0.01, 2.24)	15/13	1.94 (0.34, 10.86)	1.90 (0.34, 10.73)
*P*		0.17	0.16		0.45	0.47
Social isolation	15/13	0.49 (0.03, 4.35)	0.39 (0.04, 4.35)	15/13	0.19 (0.02, 1.10)	0.18 (0.02, 2.11)
*P*		0.45	0.44		0.17	0.17
Moderate/severe pain vs. light/no pain	14/13	6.31 (0.47, 85.40)	8.16 (0.41, 163.21)	14/13	6.31 (0.47, 85.40)	8.47 (0.44, 161.65)
*P*		0.17	0.17		0.17	0.16
Consistent pain vs. periodic/occasional/no pain	12/12	1.18 (0.20, 6.98)	1.16 (0.16, 8.62)	13/13	6.00 (0.90, 39.89)	6.00 (0.85, 42.55)
*P*		0.86	0.86		0.06	0.07
Employed	15/13	0.19 (0.02, 2.18)	^a^	15/13	0.40 (0.07, 2.42)	0.35 (0.05, 2.32)
*P*		0.18			0.32	0.28

^a^Cells too sparse to calculate an adjusted odds ratio

The results for reported pain experience were contradictory in two regression analyses. One showed that the negative impact of intervention on the SF-MPQ Pain Rating Index in the intervention group that had been observed at month 3 was no longer evident at month 6*.* In contrast, another one demonstrated that the protection effect shown at month 3 by the Wong-Baker FACES® Pain Rating Scale was gone at month 6.

The results also suggest that, 3 months after the end of the intervention, there was little difference in most of outcomes between the two groups.

## Discussion

This small pilot RCT showed that bio-psycho-social approach in this intervention had some beneficial effect on several outcomes over the period of the trial, such as handgrip strength, employment and income. A minor impact on PTSD, emotional well-being and disability score was also observed over the course of the trial, but the pattern was inconsistent.

There were some improvements in physical condition beside the changes in handgrip strength. It is known that exercise and physical activity can prevent obesity and delay the onset of various mental disturbances. Most of the traumatized participants in our trial were considered overweight or obese, and at the beginning of the trial they were inclined to be inactive and reluctant to leave their homes. It is of crucial importance that during the trial they walked out of their homes for the group therapy and exercise. A remarkable change in their physical strength was observed and reported by the therapists; i.e. at the start most of participants needed to be picked up at the main bus station in Pristina and had difficulty walking for 15–20 min to KRCT office, but at the end of the trial some of them had lost weight and some could walk from main bus station to KRCT and return to the bus station after the exhausting treatment sessions. However, both groups showed worse standing balance outcomes, which was contradictory to the therapists’ observation mentioned above with regard to their physical condition.

Based on the knowledge generated from our population-based study in Kosovo in 2008–2010, we highlighted the importance of considering the correlations among injury history, location of pain, reduction of muscle strength and other functional disability, and poor employment outcomes [1]. In the present trial we addressed these issues using a bio-psycho-social approach in our intervention, and some of the outcomes were promising.

In general, no statistically significant effect was found for mental health outcomes, although over 1/3 of PTSD cases were successfully treated and the therapists also reported noticeable clinical improvement among the participants during the trial. The lack of significant effects for most outcomes in multivariate analyses is very likely due to small size of the study population, the chronicity of physical, mental and social problems, and the very long duration of the trauma (since 1999). The PTSD score was reduced slightly at the post-treatment assessment (month 3) but a number of participants’ PTSD scores bounced back from month 3 to month 6, indicating that by the time of the follow-up the symptoms had intensified again. Half of our participants were suffering from refractory chronic pain and affective disorders, as demonstrated by the fact that they had already tried multiple pharmacological treatments and counseling without adequate or lasting effects. Comorbid refractory pain and affective disorders pose great challenges for treatment. In our study, the qualitative interview at follow-up showed that all the participants were satisfied with the treatment they had received, and they reported improvement in daily functioning, social life or found the purpose of life. Although the symptom scores remained high at the end of the trial, we do, however, consider that this type of intervention can enhance coping and physical and social functioning for people living with comorbid chronic pain and PTSD.

The problems resulting from disability should be considered in the context of the post-war economy in Kosovo. The unemployment rate in 2012 was nearly 40 % for men and 80 % for women. At the end of this trial, the employment rate among the participants was still lower than in the general population in Kosovo. However, there was a noteworthy improvement over 6 months (50 % for men and 15 % for women). The average monthly wage was 350 Euro in Kosovo in 2012, and some of participants had begun to catch up with the average wage progressively over time. By the end of the trial, there was a substantial increase in average income among those in employment, while noting that there were some missing data at the baseline. Many of the participants were from rural areas where manual labour is in demand, for which handgrip strength would be important. A study in 1999 showed that among healthy 45- to 68-year-old men, hand grip strength was highly predictive of functional limitations and disability 25 years later [46]. The increase in employment rate and in income found after the intervention could in part have been due to the improvements in physical condition simultaneously.

It is difficult to compare our results with those of earlier studies with a similar intervention approach in Europe and North America. In the early 2000s, DIGNITY (formerly Rehabilitation and Research Centre for Torture Victims) used a similar multidisciplinary approach to ours, but the patients received a larger number of treatment sessions for longer period, and the therapy was delivered by an experienced team in Copenhagen. It was reported [18, 47] that although there were no statistically significant changes in mental health outcomes from baseline to 9 months, there was a convincing improvement at a 23-month follow-up. There were also major improvements for the environmental dimension as measured by World Health Organization Quality of life, which reflected the issues from financial resources, freedom, physical safety and security, health and social care accessibility. Our study did not cover such a long time-span. The study [18, 47] was not a RCT, and outcomes of interest from other perspectives, i.e. physical examinations and employment outcome were not studied, but the participants were similar to those in our trial as they were also diagnosed with comorbid chronic pain and affective disorders before the treatment. It is interesting to see that both showed improvement potential from social perspective. Our results also support the concern that substantial symptoms remain among the refugees or immigrants with torture-related trauma in spite of treatment efforts [48], and that there was a large standard deviation for symptom reduction, which indicated a wide variation in individual outcomes and the effect of outlier when the sample size is relatively small.

It is especially important in this pilot study that the selection of outcomes involved the consideration of multiple stakeholder perspectives (patient, provider, and societal). The outcomes of this trial make us clearly aware of the complexity of the physical, psychological, familial, and social factors related to better functioning and quality of life. It also raises concerns with regard to the goals and endpoints of the treatment. Some outcome measures with functional and social perspectives showed more marked positive changes than the mental health outcome measure (illness and clinician’s perspective). This raises the question of whether the emphasis on measuring psychopathological outcomes for multidisciplinary intervention is sufficient. While evaluating impact of multidisciplinary intervention with bio-psychosocial perspective, should we not move beyond the illness-perspective and symptomatic recovery to considering broader functional recovery? If the patients regain their physical strength, search for jobs, and begin to function in daily and social life, even if pain symptoms and affective disorders remain or return, and if they develop positive coping mechanisms to handle the symptoms and the stresses of daily life, this aspect should be included in assessment of the outcomes of this type of intervention.

It is hard to compare different types of outcomes in this study, since they are based on very different measurement instruments. They have different goals, perspectives and scales, and one type of outcomes cannot be seen as a surrogate for another. In particular, it is uncertain which of the instruments are sensitive enough to detect small changes over time. Most of the research that has been done on the treatment of torture or war trauma has predominantly used subjective outcome indicators. However, these may only give one side of the picture. It is important to include objective outcome measures, like employment, income or physical strength, in similar interventions. Rehabilitation programmes have used the percentage of increase in employment in spite of disabilities as a single indicator of rehabilitation success and social reintegration. However, there are other important characteristics of a satisfactory social outcome. Stable recovery is associated with social inclusion and participation, and having a purpose in life. Without a satisfactory social outcome it is unlikely that recovery from trauma will remain stable.

It is interesting that the waiting list group also showed a reduction in symptoms while they were still waiting for treatment. However, knowing that the chronic pain and affective disorders often have a history of symptom remission and relapses, this is not unexpected. In addition, parallel improvement in several outcomes in both groups throughout the trial also suggested the potential practice effect with the same test instruments being repeatedly used over short time. The concern of practice effect needs to be addressed with chronic PTSD which is often associated with cognitive dysfunction like attention and memory. Furthermore, during the trial, the waiting list group had a realistic expectation of social support, which may give them a sense that their condition was not as bad as it used to be, so some life stressors could have been reduced. One of potential confounding factors was the intake of multivitamins. A recent study provided evidence of the beneficial effect of nutritional supplements on pain and mood status [49]. Initially, the multivitamin tablets were only given to the intervention group from the baseline. The decision to give the tablets to the waiting list participants from the beginning was to prevent drop-out due to their irritation and frustration, which were observed by the therapists. Then they became quite happy with the provision of multivitamins along. The intake of multivitamins might have created a placebo effect in this trial.

It is very difficult to control the setting of a RCT in the post-conflict context, as there were many confounding factors that we could hardly avoid. Many external factors such as dynamic social and environmental conditions may contribute to the change of symptom severity over a short time. For example, many participants were worried about their social and financial problems during the treatment and asked KRCT to help them to access official or other social support. It was observed by the therapists that some of the participants felt helpless again when the treatment terminated, which resulted in recurrence of symptoms during the follow-up.

### Strengths and limitations

One strength was that the attendance rate was high. The dropout was mainly due to illness; for example, some underwent surgery or had chemotherapy, so they could not stay in the trial since they no longer met the selection criteria. One found a full-time job and could not get the leave to continue the treatment. One of our strategies was to provide full compensation (50 Euro) for transportation and lunch if the participants attended 3 out of 4 sessions per month. Compensation was unlikely to have been a crucial incentive for taking part in a trial, since the participants could not save much from the 50 Euro they were given for expenses. The money eased the financial burden of coming to Pristina and made it possible for the participants to take part in the weekly treatment sessions without having to spend a considerable proportion of their income on paying the bus fare between their homes and Pristina, and buying lunch. The expenses were 8–10 Euro per week, which was nearly 40 % of their month’s income of around 115 Euros if they attended on weekly basis.

One weakness was the small sample size. Increase of sample size would have enhanced the generalizability and validity of the results. However, it was very difficult to approach victims a decade after the war, and persuade them to participate in the trial. Many of them did not want to open “Pandora’s box” again when they were informed about the nature of prolonged exposure therapy. Owing to the difficulty of finding more suitable participants we decided to use a minimum sample size. The length of the treatment may also have been a weakness, but in view of the local conditions, it would have been very difficult to keep the participants in a long-term treatment programme. They might have lost interest quickly if they saw no significant improvement after two or three treatment sessions. From the logistics viewpoint, we considered 10 treatment sessions to be manageable. Another shortage was that the homework component was removed from the PET protocol for the individual session. The therapists misinterpreted the homework assignment in the PET manual, in addition, the participants also felt very confused and uncomfortable with the homework in general and showed no interest in doing it.

Overall, treatment compliance and adherence to the protocol were closely monitored through supervision. However, this was the first time that DIGNITY had implemented a RCT with the partner organization in Kosovo. There were some misunderstandings of the adapted PET protocol and inconsistencies in treatment during the trial, which may have had some impact on the treatment integrity and outcome. The possibility for discrete space for conducting the homework was low in the context of Kosovo. Owing to the sensitive nature of the homework content (in vivo exposure: listening to an audio recording of treatment session or recounting aloud the full story of the trauma at home, etc.), it has been reported many times that the participants were not able to do the homework when privacy could not be ensured, since they resided in a single-room dwelling. Some participants also reported the difficulty of operating the voice recorder, and others reported their voice recorders were damaged during the study and the field team was unable to find a replacement immediately. A few of survivors of wartime rape who did not share their story with their husband worried that someone may find the tapes. Therefore, homework assignments were adjusted according to individual’s situation which confused the therapists, supervisors and the participants. However, both clinical supervisors and therapists continued to progress in learning, growing, and accumulating experience during the trial. The significance of this work was to address the implementation capacity gap in the post-conflict setting and demonstrate the Kosovo experience in building the capacity of conducting a RCT to deliver the rehabilitation service to victims, using a learning-by-doing approach and problem-driven adaption. The details of the capacity building model will be described elsewhere.

## Conclusions

In this pilot RCT, a limited effect of the intervention was observed while there were some controversies in outcomes. The promising outcomes were removal of 1/3 of PTSD cases, an increase in physical strength, employment and income, and a slight reduction in the proportion of participants with feelings of anger and hatred. We suggest that the outcome measures in a population with long-lasting effect of torture and war-related trauma should include aspects like employment, income, quality of life and his or her coping mechanisms to handle the symptoms and the daily life stressors. It is challenging to control the intervention condition and external factors in the post-war era, and adjustment to the setting as it is now will be necessary. It is recommended that treatment integrity and data reliability should be carefully monitored in order to judge the efficacy of a clinical intervention. Monitoring also enhances the trainer’s capacity to provide immediate feedback to data collectors and the therapists who are implementing procedures. Errors due to lack of treatment integrity, and to unsatisfactory data reliability, can be avoided through good training, careful description of definitions and procedures, and maintenance training. Finally, continuing social support is warranted after intervention, with the aim of preventing a return of the symptoms. The social support may include job-finding assistance, occupational training or microcredit provision, as well as various community-based activities.

## Abbreviations

BF-CBT: Biofeedback supported CBT
BMI: Body mass index
CBT: Cognitive behavioral therapy
CI: Confidence interval
DIGNITY: Danish Institute against Torture
DSM-IV: Diagnostic and Statistical Manual of Mental Disorders
HRV: Heart rate variability
HSCL-25: Hopkins symptoms checkinglsit-25
HTQ: Harvard trauma questionnaire
KRCT: Kosova Rehabilitation Centre for Torture Victims
NGO: Non-governmental organization
PET: Prolonged exposure therapy
PTSD: Post-traumatic stress disorder
RCT: Randomized controlled trial
SD: Standard deviation
SF-MPQ: Short Form McGill Pain Questionnaire
WHODAS 2.0: World Health Organization Disability Assessment Schedule 2.0 (12 Items)
